# Development of Calcium Alginate Hydrogels with *Chlorella*, Carob (*Ceratonia siliqua* L.) and Encapsulated Probiotics in Edible Jelly-Gums with Enhanced Bioactivity

**DOI:** 10.3390/gels12010001

**Published:** 2025-12-19

**Authors:** Katerina Pyrovolou, Eleni Charalampia Panopoulou, Christina Tsogka, Alexandra Sklavou, Eleni Gogou, Irini F. Strati, Spyros J. Konteles, Anthimia Batrinou

**Affiliations:** 1 Department of Food Science and Technology, University of West Attica, 12243 Athens, Greece; apyrovolou@uniwa.gr (K.P.); fst20684078@uniwa.gr (E.C.P.); fst20684105@uniwa.gr (C.T.); egogou@uniwa.gr (E.G.); estrati@uniwa.gr (I.F.S.); skonteles@uniwa.gr (S.J.K.); 2 Department of Biology, School of Science, National and Kapodistrian University of Athens, Panepistimioupolis Zografou, 15784 Athens, Greece; alexskla7@gmail.com

**Keywords:** alginate, encapsulation, *Lactobacillus acidophillus*, carob, *Chlorella*, jelly gums

## Abstract

This study aimed to develop functional calcium alginate hydrogels incorporating *Chlorella vulgaris*, carob (*Ceratonia siliqua* L.), and encapsulated *Lactobacillus acidophilus* in edible jelly gums with enhanced bioactivity and probiotic viability. Laboratory-prepared jellies containing encapsulated probiotics (encapsulated LAB-Jelly) and those with free cells (LAB-Jelly) were compared with a commercial jelly sample. The formulations were evaluated for phenolic content, antioxidant capacity and antiradical activity, texture, sensory characteristics, and probiotic survival under simulated gastrointestinal conditions. The encapsulated LAB-Jelly exhibited significantly higher total phenolic content (4.6 ± 0.1 mg GAE/g) and antioxidant activity (25.9 ± 0.1 mg Fe^+2^/g) compared to the commercial product, mainly due to the presence of carob and *Chlorella*. Texture analysis showed lower hardness (21.8 N) but comparable elasticity (89.3%) and cohesiveness (72.8%) comparative to commercial jelly gum, while sensory evaluation confirmed their favorable acceptability and non-perceptible bead presence. Microencapsulation achieved 75% efficiency and improved probiotic survival during gastrointestinal simulation after 18 h (14% reduction in Logcfu/mL compared to 30% of the free probiotics). Overall, the combination of alginate encapsulation, *Chlorella*, and carob produced edible jelly gums with improved antioxidant and textural properties, offering a promising delivery system for functional foods enriched with probiotics and plant-based bioactives.

## 1. Introduction

Probiotics are live microorganisms that, when administered in adequate amounts, confer a health benefit on the host [1]. They include bacteria, yeasts and molds and are classified as GRAS (generally recognized as safe). They can modify the intestinal mucosa, regulate the microbial balance and support immune cells, thus promoting health. In the food industry, probiotics, especially *Lactobacillus* spp. and *Bifidobacterium* spp., are naturally present in fermented foods, can be added to other food products, and are available as dietary supplements [2]. The use of probiotics has a long history, even tracing back to Ancient China. Today, the two-way connection between microbiome and health is undeniable and the possibility of controlling the latter through probiotics is under intensive research [3]. *Lactobacillus acidophilus* is a Gram-positive, non-spore-forming lactobacillus, one of the most popular probiotics worldwide. It is safe for human consumption and widely accessible. *L. acidophilus* shows high resistance to low pH values, good adhesion capacity, stability and antimicrobial activity. It has been reported to have immunomodulatory properties and acts as a metabolic regulator [4]. Despite the multiple benefits, the major problem with probiotics is their sensitivity to extreme environmental factors that makes them easily inactivated during processing, storage, and consumption [5]. As a result, one of the most complex challenges when developing probiotic products is maintaining strain’s viability during the production chain and into the gastrointestinal tract [6]. Microencapsulation, i.e., the entrapment of a compound or a system inside a dispersed material (microcapsule) for its immobilization, protection and controlled release, is a standard and common technique for ensuring probiotic viability [5]. There are several methods for creating capsules with encapsulated probiotics; one of them is extrusion, where hydrocolloid probiotic solutions are extruded through a nozzle into a hardening solution, thus turning them into gel and creating beads [7]. Extrusion is simple, easily applicable, low cost, and can employ a wide range of biopolymers as encapsulating materials. Hydrogel beads (microgels) from natural biopolymers, and especially alginate, are the most popular probiotic carriers. Alginate, an anionic polysaccharide isolated from seaweed, forms strong gels by crosslinking the anionic carboxyl groups with cationic calcium ions. It is a cheap, safe, and practical biopolymer that can be used either alone or in co-encapsulation with other biomaterials or excipients. Alginate is very stable at low pH rates and sensitive to high pH, an ideal characteristic for regulating the gastrointestinal release profiles of encapsulated molecules [8]. Despite its advantages, alginate also has some limitations: sensitivity during heat treatment, porous form, moderate stability and moderate barrier properties due to its high molecular mobility and weak interaction between molecular chains [9]. These restrictions can be overcome in many ways, like the incorporation of inorganic or organic filler materials inside the biopolymer network. Carob comes directly from carob pods (*Ceratonia siliqua* L.) after appropriate heat treatment. It is a thick, dark, aromatic, with a distinct sweet taste. It is rich in calcium, magnesium, phosphorus and sodium, an excellent plant source of sugars, proteins, fibers, vitamins and other phytonutrients. It has reported antioxidant, anti-inflammatory and antimicrobial properties, thus composing a rich functional and nutritional profile [10,11]. It is vegan, does not contain gluten or caffeine and can be used as a low-calorie sweetener [12]. The recent (re)emergence of carob-based products is not accidental. Carob combines the four main key elements of the modern food industry; it is nutritional, functional, affordable and eco-friendly [13].

*Chlorella* is a single-celled green alga with a fast growth rate and strong adaptability to different environments. It is commonly used as a functional and nutritional food additive worldwide and promotes sustainable cultivation. *Chlorella* is rich in macro- and micronutrients, most notably in high-quality protein, dietary fiber, bioactive fatty acids, vitamins and phenolic compounds [14]. Various studies suggest that *Chlorella* supports human metabolism and reports antioxidant, antidiabetic, immunomodulatory, antihyperlipidemic and antihypertensive activity [15,16]. The co-entrapment of probiotic strains with various excipients could offer better protection of the microorganisms and a final product of high bioactivity. Combinations like these can be incorporated into gummy jelly matrixes [17]. Edible jelly gums are considered particularly attractive by modern confectionery industry, because they are easy to produce, easy to consume, have pleasant texture, and can be altered accordingly to boost their nutritional and functional value without compromising their industrial and market appeal [18,19].

Existing studies have extensively explored alginate-based microencapsulation systems for the protection and delivery of probiotics and bioactive ingredients. However, the majority of previous research has focused on dairy, beverage, or pharmaceutical matrices, while only limited work has investigated their application in soft confectionery formats such as jelly gums. Furthermore, although alginate hydrogels crosslinked with calcium ions have been widely reported for their mechanical stability and controlled-release potential in biomedical and food systems [20], there is scarce published information on their integration with plant-derived functional ingredients and probiotic encapsulation simultaneously in consumer-targeted confectionery products.

This work focuses on the development of calcium alginate hydrogels combined with *Chlorella*, carob and encapsulated *Lactobacillus acidophilus* in edible jelly gums with enhanced bioactivity. Our results are based on microbiological and physicochemical analyses and could contribute to the creation of novel, more effective and functional probiotic products.

## 2. Results and Discussion

### 2.1. Spectrophotometric Assays

**Figure 1:**
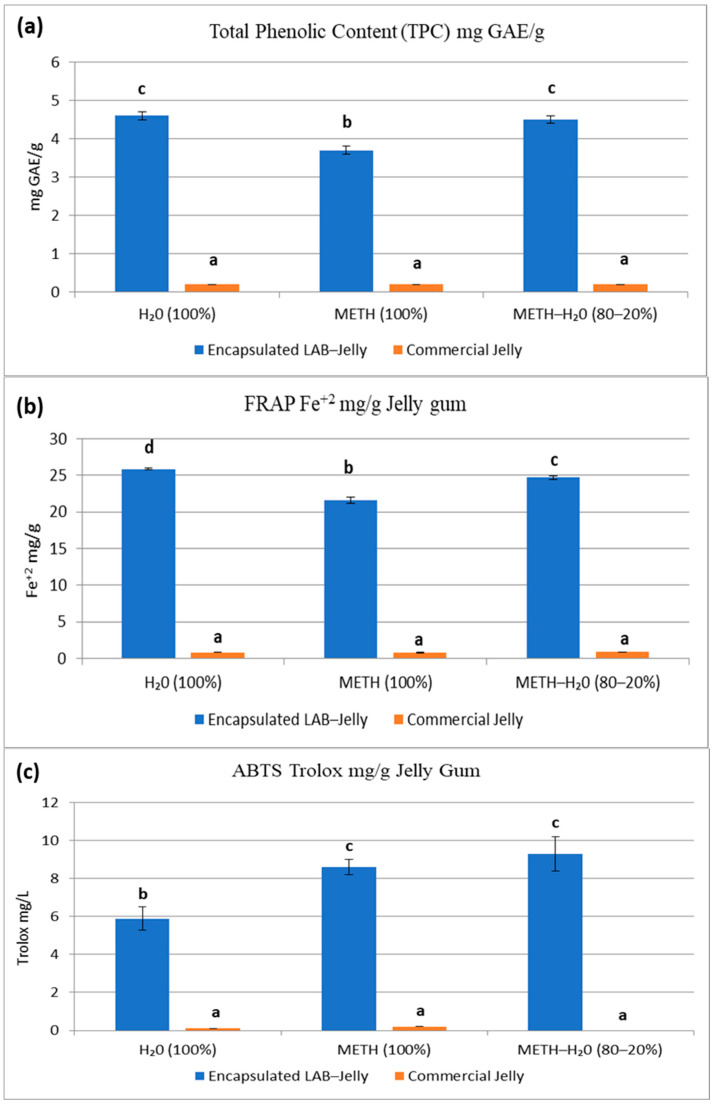
(**a**) Total phenolic content of encapsulated LAB-Jelly and commercial Jelly gums. (**b**) Antioxidant capacity of encapsulated LAB-Jelly and commercial Jelly gums. (**c**) Antiradical activity of encapsulated LAB-Jelly and commercial Jelly gums. Bars bearing different letters are statistically different (*p* < 0.05) (a < b < c < d).

According to bibliographic references, carob is considered a rich source of several bioactive compounds beneficial to human health, including minerals and phenolic constituents [21]. Phenolic compounds are secondary metabolites produced by plants and are present in all plant-derived materials [22]. In the case of carob (*Ceratonia siliqua* L.), the main subclasses of polyphenols include phenolic acids, flavonoids, and tannins [23]. As natural antioxidants, polyphenols protect cells from oxidative damage, while carob has been reported to exhibit anticancer, antidiabetic, and neuroprotective properties [23]. In this study, the extraction of antioxidant compounds was performed with three different solvents: 100% water, methanol/water (80:20) and 100% methanol since jelly gums are complex matrices containing hydrophilic and hydrophobic ingredients (e.g., simple and complex sugars, pectin, proteins, etc.) and the choice of extraction solvent may influence the quantification of antioxidant compounds and the resulting antioxidant capacity. The analysis of Total Phenolic Content (TPC) in jelly samples extracted in three different solvents revealed that water was an effective solvent for both samples (encapsulated LAB-Jelly and commercial jelly) which is an important finding since the dissolution of jelly gums in water resembles more the physiological conditions (dissolution in the GI tract) (Figure 1a). The non-food grade methanol-water (80:10) mixture was also efficient in extracting polyphenols. A clear difference was observed (Figure 1a) between the two jelly gum samples regarding their total phenolic content (TPC). The laboratory-prepared jelly gum had a total phenolic content of 4.6 ± 0.08 mg GAE/g jelly gum (in water extract), whereas the commercial jelly had 0.2 ± 0.03 mg GAE/g. This variation can be attributed to the composition of the formulations, as carob was the predominant ingredient in the encapsulated LAB-Jelly gums, whereas sugar constituted the main component of the commercial samples. According to the literature, carob (*Ceratonia siliqua* L.) is known to be rich in polyphenolic compounds, which significantly contribute to the higher TPC values observed [21]. Moreover, the presence of *Chlorella* in the formulation may have also influenced the final phenolic content due to its own antioxidant potential [24]. Similar studies have determined a mean value of 9.51–14.80 mg GAE/g among nine carobs [23], 351.4 ± 4.1 mg GAE/100 g carob fruit by Goulas and Georgiou (2020) and 11.57 mg GAE/g extract by Gregoriou et al. (2021) [25,26].

Concerning the antioxidant activity assessed by FRAP Assay, water was the most effective solvent for both samples (Figure 1b) and the encapsulated LAB-Jelly gum exhibited antioxidant capacity (25.9 ± 0.09 mg Fe^+2^/g) more than 20 times higher than the commercial jelly (0.8 ± 0.01 mg c^+2^/g). This can be explained by the presence of carob and *Chlorella* which have enhanced the antioxidant capacity, given their high content of bioactive compounds with antioxidant properties [27,28]. In the antiradical assay (ABTS^•+^), methanol–water (80:20) solvent mixture appeared to be the most effective solvent for the encapsulated LAB-Jelly gum. A notable significant difference was observed (Figure 1c) between the two jelly gum samples in terms of their antiradical activity. The encapsulated LAB-Jelly gum possessed antiradical activity expressed as 9.3 ± 0.89 mg Trolox Equivalents (TE)/g jelly gum, whereas the commercial jelly had 0.2 ± 0.01 mg TE/g jelly gum.

### 2.2. Texture Analysis

**Figure 2:**
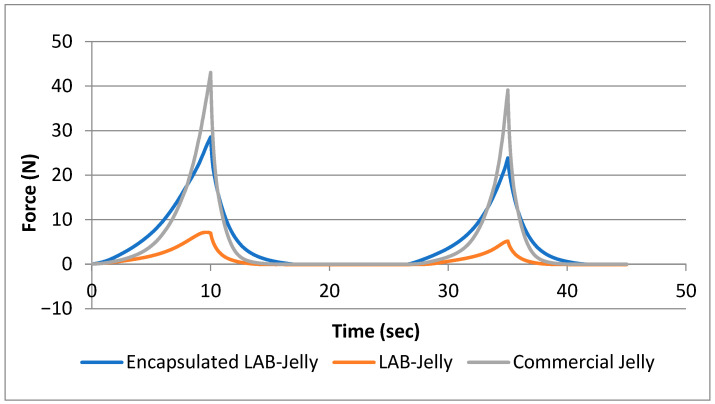
Comparative Texture Profile Analysis (TPA) graph of the Three Samples.

Notable differences were observed among the three samples in terms of their textural parameters (Figure 2). Specifically, hardness which represents the force required for the first bite was considerably higher in the commercial Jelly compared to both laboratory-prepared samples (encapsulated LAB-Jelly and LAB-Jelly). This can be attributed to the different formulations of jellies when compared to commercial and possibly differences in water content. More specifically, the commercial jelly was tested in its original form to reflect realistic market conditions, although its texture was considerably harder due to its low water activity (aw ≈ 0.4) compared with the laboratory-produced jelly gums (aw ≈ 0.97). Additional textural differences may also be attributed to ingredients in the commercial product, such as carnauba wax, which is commonly used to create protective surface coatings. As expected, the encapsulated LAB-Jelly exhibited greater hardness than LAB-Jelly, due to the presence of the encapsulating beads [29]. Springiness, which reflects the ability of the sample to recover its original shape after the first compression, did not show significant differences among the three samples. This is desirable for the texture of the encapsulated LAB-Jelly, as it indicates characteristics consistent with commercially acceptable products [30]. Regarding cohesiveness, which is associated with the structural integrity of the sample during mastication [31], similar values were also recorded for all three samples. The LAB-Jelly sample showed the lowest cohesiveness, which can be explained by the higher liquid content resulting from the addition of free probiotic cells during preparation. Finally, adhesiveness, describing the stickiness of the product during chewing [31], presented comparable values across all samples, even though commercial Jelly contained a much higher sugar content than the laboratory-prepared samples.

**Figure 3:**
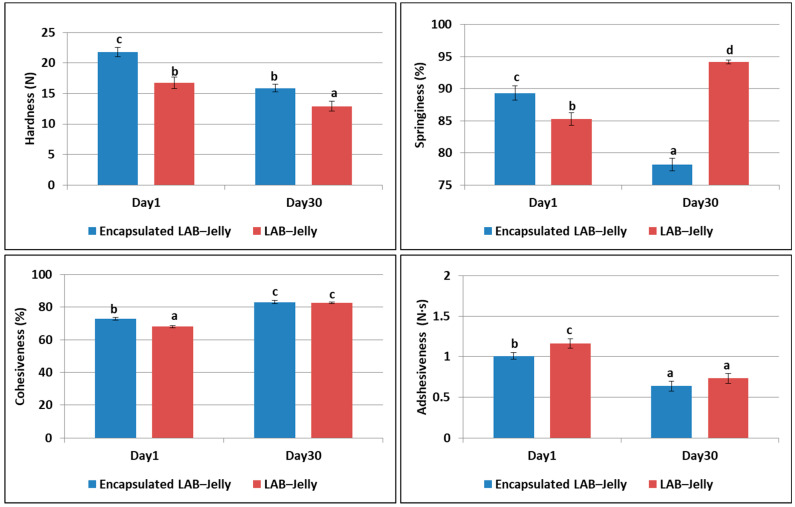
Evaluation of textural properties after one month refrigerated storage of encapsulated-LAB-Jelly and LAB-Jelly gums. Bars bearing different letters are statistically different (*p* < 0.05) (a < b < c < d).

The laboratory-prepared jelly gums were re-evaluated after one month of storage at refrigeration temperature, and the corresponding results are summarized in Table 1. Hardness decreased in all samples, with the encapsulated LAB-Jelly decreasing from 21.8 N to 15.9 N, and the LAB-Jelly from 16.7 N to 12.9 N, indicating a softening of the structure, likely due to moisture absorption. However, considering the standard deviation (Figure 3), the differences were relatively small. Regarding springiness, the encapsulated LAB-Jelly showed a decrease (89.3% to 78.2%), whereas an increase was observed in LAB-Jelly (85.3% to 94.2%). This increase is likely related to moisture redistribution within the gel matrix. Cohesiveness showed an upward trend in both the encapsulated LAB-Jelly (72.8% to 83.1%) and the LAB-Jelly (68.1% to 82.6%), indicating a strengthening of the internal structure over time. Finally, adhesiveness decreased in the encapsulated LAB-Jelly (1.00 to 0.64 N·s) and the LAB-Jelly (1.17 to 0.73 N·s), implying a reduction in stickiness during storage, possibly due to moisture retention within the gel matrix. Overall, the encapsulated LAB-Jelly exhibited smaller textural changes during storage, suggesting enhanced texture stability compared to LAB-Jelly. In conclusion, the microencapsulation technology positively influences the texture of jelly gums since the presence of beads improves hardness, springiness, and cohesiveness, making the laboratory-prepared samples more comparable to the commercial Jelly, while simultaneously reducing adhesiveness. In contrast, LAB-Jelly exhibited inferior textural characteristics, which may negatively affect consumer acceptability. However, this study was exploratory and conducted at laboratory scale and future studies should use power analysis to ensure statistical robustness.

Although there are limited data for texture analysis in jelly gums, in a comparable study on jelly candies, the corresponding texture parameters were reported to be 24.5 N for hardness, 89.3% for springiness, 76.3% for cohesiveness, and 0.16 N·s for adhesiveness [31].

### 2.3. Sensory Evaluation

**Figure 4:**
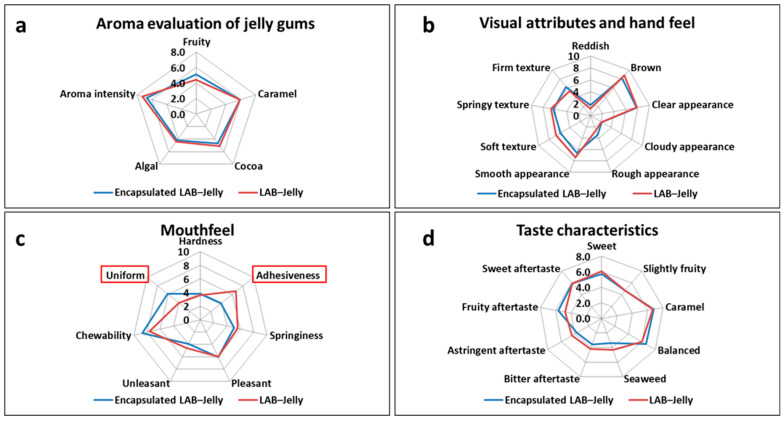
Sensory Evaluation Results of encapsulated LAB-Jelly and LAB-Jelly gums, (**a**) Aroma evaluation of Jelly gums, (**b**) Visual attributes and hand feel, (**c**) Mouthfeel, (**d**) Taste characteristics. Red frames indicate attributes with statistically significant differences.

The results of the sensory evaluation of the jelly gums are presented in Figure 4. Regarding the aroma evaluation (Figure 4a), it can be observed that no statistically significant differences were detected among the assessed attribute, as the two samples (encapsulated LAB-Jelly and LAB-Jelly) were prepared using the same ingredients in identical proportions. Similarly, Figure 4b confirms that no differences were found between the two samples in terms of texture and appearance. Regarding texture, minor variations were noted, which can be attributed to the addition of 10 mL of diluent in the sample in order to incorporate the free probiotics. In contrast, Figure 4c shows statistically significant differences in uniformity of mastication and adhesiveness. The encapsulated LAB-Jelly exhibited higher scores for uniform mastication and lower scores for adhesiveness compared to the LAB-Jelly, indicating a potential advantage in consumer acceptability. Finally, taste attributes (Figure 4d) did not differ significantly between the samples, as both were prepared using the same formulation. In conclusion, the encapsulated LAB-Jelly was evaluated as technologically and organoleptically satisfactory, being more acceptable to the panelists. The presence of the beads was not perceptible, suggesting that they can be incorporated into the product without negatively affecting sensory perception, thus supporting their use in functional confectionery formulations. However, the sensory evaluation performed was an initial sensory screening and further acceptance testing with a larger, demographically diverse group is required to establish market potential.

### 2.4. Microbiological Analysis Results

Microbiological analysis was performed at all stages of the production process—from the inoculum to the final product for the encapsulated LAB-Jelly. As shown in Table 2, the bacterial population decreased by approximately one logarithmic cycle at each stage of the production process. All preparations were performed under aseptic conditions and the decrease in viable counts most probably corresponded to handling losses during centrifugation, continued dilution, and bead formation steps in line with previous reports [32].

The encapsulation efficiency (EE%) measured as the ratio of the bacterial population after microencapsulation (N_2_) to the bacterial population of the inoculum (N_0_) was 75% which is considered satisfactory [33].

The probiotic population of both the encapsulated LAB-Jelly and the free LAB-Jelly was assessed after 30 days of storage at 4 °C in Petri dishes. The viable counts were 6.5 and 6.4 log CFU/mL, respectively, indicating satisfactory survival during refrigerated storage. In addition, all samples were tested for yeast and molds on day 1 of production and again after 30 days of storage at 4 °C, with negative results in both cases. However, the authors acknowledge that a more comprehensive evaluation of product stability and pathogen testing is needed, particularly given the high water activity of the current formulation (aw ≈ 0.97). Future work will focus on optimizing processing and formulation strategies to reduce aw to levels that enhance microbiological stability while maintaining the viability of the incorporated probiotics.

### 2.5. In Vitro Gastrointestinal Simulation

**Figure 5:**
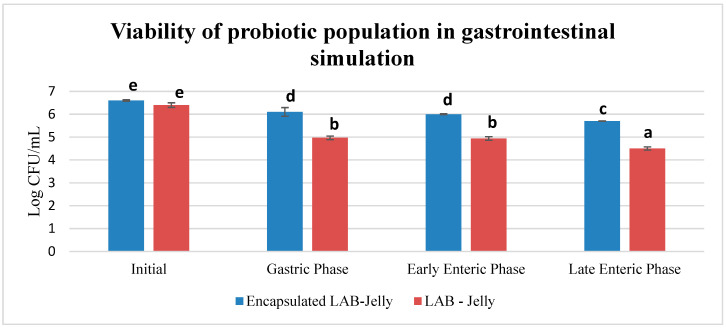
Viability in Log CFU/mL of *L. acidophilus* after gastrointestinal simulation (GI). Bars bearing different letters are statistically different (*p* < 0.05) (a < b < c < d < e).

According to the obtained results (Figure 5), microencapsulation proved to be overall effective in maintaining probiotic viability under the harsh conditions of the gastric phase. Regarding the simulated gastrointestinal (GI) digestion process, a higher survival rate was observed at all stages for the jelly gums containing encapsulated probiotics compared to the control sample with free cells (Table 3). At the final stage of GI simulation, the reduction in viability of the encapsulated probiotics was <1 log and 14% expressed as % reduction whereas the reduction in the free probiotics was almost 2 logs (1.9) and 30%. This finding demonstrates the protective effect of microencapsulation against the adverse conditions of the gastrointestinal environment. The commercial product was tested in the static GI simulation system, in which three jelly gums (~6 g total) were used for each phase (initial, gastric, enteric, and late enteric phase). No colonies were detected on MRS agar and anaerobic incubation at any stage. This procedure was intended to mimic real product consumption in order to assess the number of viable probiotics after each phase of gastrointestinal transit. However, given the unexpected result of zero growth, a further evaluation was conducted in which three commercial jelly gums were transferred aseptically into MRS broth and incubated anaerobically for 48 h. After this stage of enrichment, the results showed that the viable probiotic population reached 9.3 log CFU/g. This indicates that the probiotic cultures of the commercial product may be at a lethargic state or a loss of cell viability has occurred during processing and storage. In comparable gastrointestinal simulation studies, the viability of free and encapsulated probiotics after 2 h of incubation (GF) was reported to be 67.2% and 92.1%, respectively [34].

## 3. Conclusions

In this study, the production of jelly gums with microencapsulated probiotics (*Lactobacillus acidophilus*) was investigated, using sodium alginate as the coated material. The experimental results demonstrated that microencapsulation played a decisive role in maintaining probiotic viability, reducing the Log bacterial population by 14% compared to 30% in free probiotic samples after exposure to simulated gastrointestinal conditions. The use of carob syrup proved particularly beneficial due to its high content of polyphenols and antioxidants. Comparative experiments performed in the laboratory revealed significant differences in phenolic content, antioxidant and antiradical activity compared to commercial samples, with values of 4.6 mg GAE/g, 25.9 ± 0.09 mg Fe^+2^/g, and 9.3 ± 0.89 mg Trolox Equivalents (TE)/g, respectively, which were more than 20 times higher than the commercial Jelly. Furthermore, texture showed lower hardness (21.8 N) but comparable elasticity (89.3%) and cohesiveness (72.8%) comparative to commercial Jelly. Despite the promising results, this study presents certain limitations that should be acknowledged. First, the experiments were conducted on a laboratory scale, which may not fully reflect the performance or stability of the product under industrial processing conditions. Furthermore, while extrusion is suitable for small-scale production, larger scale production will require optimization and validation and may be the use of other industrial alternatives (e.g., jet cutting). Additionally, the storage study was limited to one month under refrigeration, and therefore long-term stability, shelf life, and potential changes in probiotic viability or textural properties remain uncharacterized. Recommendations for future work include shelf life testing, formulation optimization and compositional characterization, study of separate matrix effects on texture and bioactivity, gel-network analysis, sensory testing on larger and more demographically diverse populations, evaluation of viability in semi-dynamic or dynamic digestion models, cost analysis, and scale-up feasibility.

In conclusion, this study highlighted the potential of microencapsulation technology for the protection of probiotics and the functional contribution of natural ingredients such as carob and *Chlorella* in the development of a nutritionally enhanced functional product. The findings establish a foundation for further research and development of next-generation functional foods that can effectively contribute to improved health and the promotion of a more balanced diet.

## 4. Materials and Methods

### 4.1. Preparation of Samples

#### 4.1.1. Types of Samples

Two types of samples were prepared in the laboratory: (a) the “encapsulated LAB-Jelly gums” which were jelly samples with the incorporation of alginate beads containing the probiotic culture and all the other ingredients as mentioned in Section 4.1.4 and (b) the “LAB-Jelly gums” which were also laboratory-prepared, but the probiotic inoculum was added directly into the jelly matrix without encapsulation, as “free” cells. Additionally, commercial jelly gum containing free (non-encapsulated) probiotics, purchased from a local pharmacy was used for the analyses. The commercial jelly was selected as one of the “best sellers” in the market according to e-commerce sites and consisted of glucose syrup, sugar, pectin, sodium citrate, maltodextrin, colorant, carnauba, flavoring, nicotinamide, vitamins B5 and B6. The package claimed to have a population of 10^9^ viable probiotic bacteria (*Lactobacillus rhamnosus* and *Bifidobacterium infantis*) and it had a 12-month shelf life. For the spectrophotometric analyses and sensory evaluation, the samples used were encapsulated LAB-Jelly and commercial Jelly. For the microbiological analyses and texture analysis, the samples used were encapsulated LAB-Jelly, LAB-Jelly, and commercial Jelly.

#### 4.1.2. Preparation of Probiotic Inoculum

*Lactobacillus acidophilus* LA85 was used as probiotic inoculum. The strain was initially cultured in 9 mL of MRS broth at 37 °C for 48 h. After thorough mixing, the culture was transferred to 50 mL sterilized Falcon tubes (Merck KGaA, Darmstadt, Germany) then sterilized MRS broth (Condalab, Madrid, Spain) was added to reach a total volume of 40 mL and cultures were incubated at 37 °C for 48 h. The cultures were then collected by centrifugation using a Sorvall RC-3B Superspeed Centrifuge (Thermo Scientific, Waltham, MA, USA) at 4500 rpm for 15 min and the supernatant was discarded. The final inoculum volume, after combining all Falcon tubes, was 10 mL.

#### 4.1.3. Microencapsulation of Lactobacillus Acidophilus LA85

**Figure 6:**
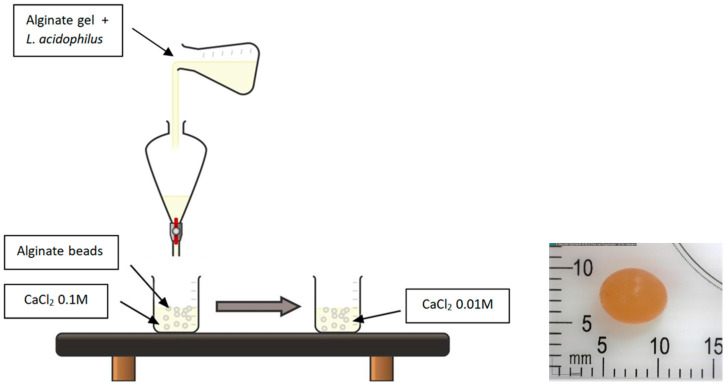
Microencapsulation of *Lactobacillus acidophilus* LA85 in spherical alginate beads. In the photo right, one individual bead is shown.

100 mL of 2% sodium alginate gel was prepared by dissolving 2.2 g of sodium alginate powder in 100 mL of distilled water with constant stirring and mild heating. 10 mL probiotic inoculum as prepared in Section 4.1.2 was then added to the alginate gel, and the mixture was transferred to a funnel to initiate bead formation by extrusion in a 0.1 M CaCl_2_ solution. The funnel was placed at 72 cm above the collection beaker, and the rate of extrusion was set at 22 beads/min. The beads were subsequently transferred to CaCl_2_ 0.01 M solution for 30 min to harden and were stored in a sterile container under refrigerated conditions (Figure 6). All procedures were conducted under aseptic conditions. The average bead diameter was 5.3 ± 1.3 mm (analysis performed via imaging with Dino Lite Microscopy, Taipei, Taiwan).

#### 4.1.4. Preparation of Encapsulated LAB–Jelly

**Figure 7:**
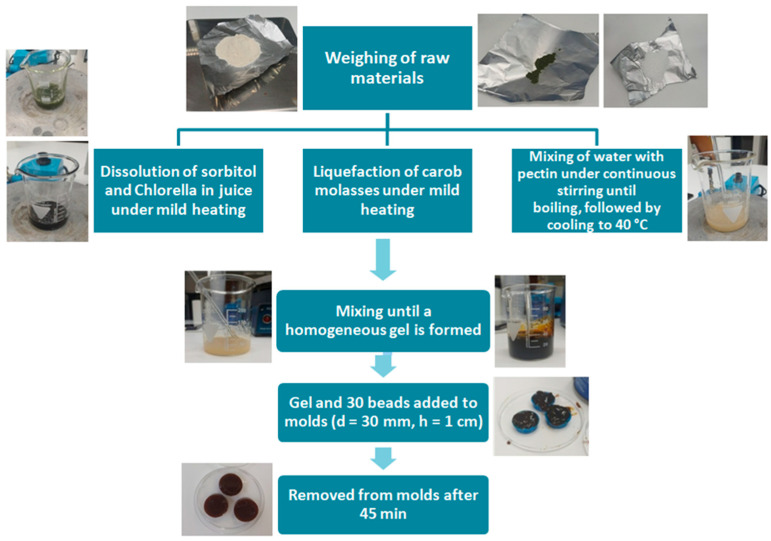
Flow chart for the preparation of encapsulated LAB-Jelly.

The jelly was prepared by mixing 17 g of peach–apricot juice purchased from a local supermarket, 20 g carob syrup from Cretan carobs (kindly provided by RAKS Petrakis, Athens, Greece), 5 g pectin (Grindsted Pectin LA 410, Danisco, Smirice, Czech Republic), 0.06 g *Chlorella* (Health Trade, Athens, Greece), 1.4 g sorbitol (Serva, Heidelberg, Germany) and 30 g water. After weighing all ingredients in a beaker, water was mixed with pectin under continuous stirring and heating until the mixture reached boiling and became homogeneous (Figure 7). Simultaneously, the carob syrup was slightly heated in a separate beaker until liquefied, while in another beaker the juice and sorbitol were warmed to reach approximately the same temperature. The pectin mixture was then removed from the heating plate, and the carob, fruit juice–sorbitol mixture, and *Chlorella* were added. Once all components reached a similar temperature, they were thoroughly mixed until a homogeneous mixture was obtained. The final mixture was transferred into 3D-printed molds with a diameter of approximately 30 mm and a height of 1 cm and the alginate beads with the probiotics were added to form the “encapsulated LAB-Jelly” (Figure 7). After 45 min, the jelly gums were removed from the molds and stored under refrigeration.

The LAB-Jelly was prepared similarly to the encapsulated LAB-Jelly, with the only difference being the mixing step. In this step, in addition to the other ingredients, 10 mL of diluent containing a probiotic inoculum of approximately the same population as the encapsulated probiotic was added. Then, the mixture was transferred to molds and allowed to set for 45 min.

### 4.2. In Vitro Gastrointestinal Simulation

**Figure 8:**
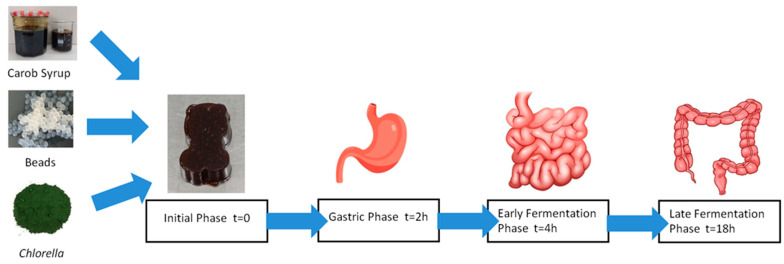
Gastrointestinal simulation in a static three-phase system.

The gastrointestinal simulation followed a standardized static in vitro digestion model. The enzyme concentrations and activities (pepsin, pancreatin, and lipase) were prepared according to established protocols and verified prior to use. Gastric and intestinal fluids were prepared according to the method described by Paramera et al. (2011) [35]. For the gastric fluid, all components were dissolved and the pH was adjusted to 2 using HCl 0.1 N. For the intestinal fluid, pancreatin (Pancreatin PanReac Applichem, Barcelona, Spain (activity: 36,000 FIP U/g, 0.1% *w*/*v*) and bile salts (Bile extract porcine Sigma, St. Louis, MO, USA, 0.3% *w*/*v*), were added, and the pH was adjusted to 7 using NaOH 0.1 N. All samples (encapsulated LAB-Jelly, LAB-Jelly, and commercial Jelly) were weighed, and ten times the amount of gastric fluid was added. A three-phase static model (Figure 8) was used in which samples were incubated in the gastric fluid at 37 °C for 2 h (Gastric phase) under constant agitation, then transferred to the intestinal fluid additionally for 2 h (Early Enteric Fermentation phase), followed by further incubation in the intestinal fluid for 12 h (Late Enteric Fermentation phase). Microbial counts were evaluated at 0, 2, 4 and 18 h.

### 4.3. Spectrophotometric Assays

For all assays, the samples were dissolved in three different solvent mixtures: (a) 100% water, (b) methanol/water (80:20) and (c) methanol 100%. These solvents with different polarities (highly polar to lower polar) were used in order to evaluate their efficiency in extracting the antioxidant compounds of the jelly gums since there were limited published data regarding optimal solvents for analyzing jelly gums.

#### 4.3.1. Determination of Total Phenolic Content (TPC)

The total phenolic content (TPC) of the jelly gums was determined using a modified version of the Folin–Ciocâlteu assay [36]. For the encapsulated LAB-Jelly, 30.0 μL of the sample, 2470.0 μL of distilled H_2_O, and 200.0 μL of Folin–Ciocâlteu reagent were added to plastic cuvettes. After thorough mixing, the samples were kept in the dark for 8 min. Subsequently, 500.0 μL of a saturated Na_2_CO_3_ solution was added, followed by further mixing. The cuvettes were then incubated in a water bath at 40 °C for 30 min, protected from light. After color development (formation of the characteristic blue coloration), absorbance was measured at 750 nm at room temperature using a Vis spectrophotometer (Spectro 23 Digital Spectrophotometer, Labomed Inc., Culver City, CA, USA). Results were expressed as mg gallic acid equivalents (GAE) per g of sample, based on a standard curve prepared with gallic acid solutions with 5–500 mg/L (y = 0.0035x + 0.0132, R^2^ = 0.9991). For the determination of the total phenolic content of the commercial jelly gum sample, the same experimental procedure was followed, with the modification that 200.0 μL of the sample and 2300.0 μL of distilled H_2_O were used.

#### 4.3.2. Ferric Reducing/Antioxidant Power (FRAP) Assay

The ferric reducing antioxidant power (FRAP) assay was used to evaluate the ability of samples to reduce Fe(III) to Fe(II) [37]. For the encapsulated LAB-Jelly gum, 30.0 μL of the sample, 1970.0 μL of distilled H_2_O, 900.0 μL of FRAP reagent, and 500.0 μL of buffer solution were added to plastic cuvettes. The mixture was stirred and incubated in a water bath at 40 °C for 90 min. Absorbance was measured at 595 nm using a Vis spectrophotometer (Spectro 23 Digital Spectrophotometer, Labomed Inc., Culver City, CA, USA). Results were expressed as mg Fe(II) per g of jelly gum, using a standard curve prepared with FeSO_4_·7H_2_O solutions with a range of 50–1800 μM (y = 0.000x + 0.008, R^2^ = 0.996). For the determination of the antioxidant capacity of the commercial jelly gum sample, the same experimental procedure was followed, with the modification that 200 μL of sample and 1800 μL of distilled H_2_O were used.

#### 4.3.3. Scavenging Activity on 2,2′-Azino-bis-(3-ethylbenzothiazoline-6-sulfonic Acid) Radical (ABTS^•+^)

The radical scavenging activity of the samples was determined according to the method described by Miller et al. (1993) [38], which is based on a decolorization reaction. The blue-green ABTS^●+^ radical cation is generated by the oxidation of the non-radical ABTS molecule with potassium or sodium persulfate. In the presence of hydrogen-donating compounds, the ABTS^●+^ radical is reduced in proportion to the hydrogen-donating capacity, the concentration of the donor molecule, and the reaction time [39]. The ABTS^●+^ stock solution was prepared by reacting 7 mM ABTS with 2.45 mM sodium persulfate (Na_2_S_2_O_8_) in a volume ratio of 1.0:0.5, and the mixture was incubated in the dark at room temperature for 16 h. Prior to use, an 8.5 mM Trolox solution was diluted with 90 mL of ethanol to achieve an absorbance of 0.7–0.8 at 734 nm. For the encapsulated LAB-Jelly gums, 20.0 μL of the sample and 3000.0 μL of the diluted ABTS^●+^ solution were added to plastic cuvettes, followed by mixing. After 8 min, absorbance was measured at 734 nm using a Vis spectrophotometer (Spectro 23 Digital Spectrophotometer, Labomed Inc., Culver City, CA, USA). Results were expressed as mg Trolox equivalents (TE) per L of sample, based on a calibration curve constructed with Trolox concentrations ranging from 0.2 to 1.5 mM (y = 0.2876x − 0.002, R^2^ = 0.999). For the determination of the antioxidant capacity of the corresponding commercial jelly gum sample, the same experimental procedure was followed, with the modification that 200 μL of sample.

### 4.4. Texture Analysis

Texture analysis was performed using a texture analyzer with a load cell of 5 kg (TAX.TX Plus, Stable Micro Systems, Surrey, UK). Samples were subjected to Double Compression test to construct the Texture Profile Analysis (TPA) graphs, using a clear acrylic cylinder probe of 25 mm diameter at a test speed of 0.5 mm/s, with a 5 s interval between the two compression cycles. Force–time (N–s) curves were recorded and analyzed using the Texture Exponent version 32 Application software (Stable Micro Systems Ltd., UK). The following texture parameters were calculated and compared: hardness, springiness, cohesiveness, and adhesiveness. Measurements were conducted on the jelly gum samples at two different time points (Day 1 and Day 30) in order to evaluate changes in texture during storage under refrigerated conditions (4 °C in Petri dish). A control jelly containing *Chlorella* and carob but without added probiotics was not included in the present work; however, this comparison will be incorporated in future experiments to isolate more clearly the specific contribution of the microencapsulated probiotics to the product’s texture and sensory profile.

### 4.5. Sensory Evaluation

The sensory evaluation was conducted by a panel of 18 untrained assessors, consisting of students and staff members from the Department of Food Science and Technology at the University of West Attica. The samples evaluated were the encapsulated LAB-Jelly (coded Sample 1) and the LAB-Jelly (coded Sample 2) gums. The objective of the evaluation was to identify differences in appearance, texture, flavor, and overall acceptability between the products. The assessment included parameters related to color, smell, texture, chewiness, and aftertaste. Each sensory attribute was rated on a 10-point scale (1 = lowest intensity/not perceptible; 10 = highest intensity/extremely perceptible). Data were analyzed using analysis of variance (ANOVA) at a significance level of *p* < 0.05. During the evaluation, bottled water was provided to the panelists for palate cleansing between samples.

### 4.6. Microbiological Analysis

#### 4.6.1. Lactobacillus Acidophilus Inoculum/Alginate Gel

A 1 mL aliquot was taken and transferred into diluent for serial dilutions. From each dilution, samples were plated onto MRS agar using the incorporation method and incubated under anaerobic conditions at 37 °C for 48 h.

#### 4.6.2. Alginate Beads

For the beads, 1 g of sample was weighed into a stomacher bag, and 10 mL of trisodium citrate was added. The mixture was processed in a stomacher until complete dissolution. The resulting solution was then used for serial dilutions and plating onto MRS agar, following the same procedure as described previously.

#### 4.6.3. Jelly Gums

Microbiological analyses were performed on all samples of jelly gums. For the encapsulated LAB-Jelly, a jelly gum was weighed, and ten times its weight of trisodium citrate was added. For the LAB-Jelly and commercial jelly gums, ten times their weight of diluent was added. The same procedure was then followed, including serial dilutions and plating onto MRS agar. Microbiological analyses were performed on all samples, at the end and during the gastrointestinal simulation. For the encapsulated LAB-Jelly, after 2 h, the gastric fluid was removed, and ten times the sample weight of trisodium citrate was added to dissolve the beads. Serial dilutions were then prepared, and aliquots were plated onto MRS agar. At 4 h and 18 h, since both the jelly gum and the beads had been dissolved, trisodium citrate was added to the sample, followed by serial dilutions and plating. The same procedure was applied to the LAB-Jelly and commercial Jelly at the same time points, except that trisodium citrate was not used due to the absence of beads. All plates were incubated under anaerobic conditions at 37 °C for 48 h.

#### 4.6.4. Yeasts and Molds

Microbiological analysis of yeasts and molds was performed in encapsulated LAB-Jelly and LAB-Jelly. Serial decimal dilutions of samples were prepared in aseptic conditions and 0.1 mL of each dilution was spread with a Drigalski spatula on the surface of Sabouraud Dextrose Agar plates. The plates were incubated aerobically on 25 °C for 5 days.

### 4.7. Water Activity

Water activity was measured using the AQUALAB 4TE (AquaLab, Pullman, WA, USA), water activity meter. The aw values of all three samples were determined at two time points, on day 1 and day 30. Each sample was placed in the instrument, and after an equilibration period of approximately 15 min, the corresponding water activity value was recorded.

### 4.8. Statistical Analysis

All analyses were performed in triplicates and standard deviations were calculated. Statistical analysis was performed by IBM SPSS 29.0 (IBM Corp., Armonk, NY, USA).

## Figures and Tables

**Table 1:** Comparative texture profile analysis results and percentage difference in tested Jelly gums (Day 1–Day 30). Different letters are statistically different (*p* < 0.05) (a < b < c < d).

	Textural Properties	Day1	Day30	% Difference
Encapsulated LAB-Jelly	Hardness (N)	21.8 ^c^	15.9 ^b^	27.2
	Springiness (%)	89.3 ^c^	78.2 ^a^	12.4
	Cohesiveness (%)	72.8 ^b^	83.1 ^c^	14.2
	Adhesiveness (N·s)	1.00 ^b^	0.639 ^a^	36.2
LAB-Jelly	Hardness (N)	16.7 ^b^	12.9 ^a^	22.8
	Springiness (%)	85.3 ^b^	94.2 ^d^	10.4
	Cohesiveness (%)	68.0 ^a^	82.6 ^c^	21.4
	Adhesiveness (N·s)	1.17 ^c^	0.734 ^a^	37.2
Commercial Jelly	Hardness (N)	43.1	No data	
	Springiness (%)	90.8	No data	
	Cohesiveness (%)	75.9	No data	
	Adhesiveness (N·s)	1.00	No data	

**Table 2:** Microbiological analysis of encapsulated LAB-Jelly and microencapsulation efficiency.

Sample	Encapsulated LAB-Jelly (Log CFU/mL)	%EE ^5^
^1^ N_0_	9.6	75%
^2^ N_1_	8.6	
^3^ N_2_	7.2	
^4^ N_3_	6.6	

^1^ N_0_: bacterial population of the inoculum, ^2^ N_1_: bacterial population in the alginate gel after inoculum addition, ^3^ N_2_: bacterial population after microencapsulation, ^4^ N_3_: Bacterial population of the encapsulated LAB–Jelly (expressed in log CFU/mL), ^5^ EE%: Encapsulation Efficiency (%).

**Table 3:** Survival rate of laboratory-prepared jelly gums after gastrointestinal simulation (GI).

Phases in GI Simulation	Encapsulated LAB-Jelly (LogCFU/mL)	* %Reduction in Viability	LAB-Jelly (LogCFU/mL)	* %Reduction in Viability
Initial (t = 0 h)	6.60		6.40	
GastricPhase (t = 2 h)	6.10	8%	4.97	22%
Early Enteric Phase (t = 4 h)	6.00	9%	4.94	23%
Late Enteric Phase (t = 18 h)	5.70	14%	4.50	30%

* Reduction in Viability (%) was calculated at each phase as ((log N_initial_ − log N_final_)/log N_initial_) × 100, where N_final_ is the CFU/mL of any phase and N_initial_ is the CFU/mL of the initial phase, representing the % reduction in log number of probiotic cells (encapsulated and free) after exposure to simulated gastrointestinal conditions.

## Data Availability

The original contributions presented in this study are included in the article. Further inquiries can be directed to the corresponding author.
